# Mast Cell Tryptase Promotes Airway Remodeling by Inducing Anti-Apoptotic and Cell Growth Properties in Human Alveolar and Bronchial Epithelial Cells

**DOI:** 10.3390/cells12101439

**Published:** 2023-05-22

**Authors:** Frida Berlin, Sofia Mogren, Camilla Ly, Sangeetha Ramu, Morten Hvidtfeldt, Lena Uller, Celeste Porsbjerg, Cecilia K. Andersson

**Affiliations:** 1 Department of Experimental Medical Science, Lund University, 22184 Lund, Sweden; 2 Department of Respiratory Medicine, Copenhagen University Hospital Bispebjerg, 2400 Copenhagen, Denmark

**Keywords:** mast cell, proteases, tryptase, bronchial epithelium, alveolar epithelium, growth factors, anti-apoptosis, airway remodeling, cell growth, PAR-2

## Abstract

Bronchial and alveolar remodeling and impaired epithelial function are characteristics of chronic respiratory diseases. In these patients, an increased number of mast cells (MCs) positive for serine proteases, tryptase and chymase, infiltrate the epithelium and alveolar parenchyma. However, little is known regarding the implication of intraepithelial MCs on the local environment, such as epithelial cell function and properties. In this study, we investigated whether MC tryptase is involved in bronchial and alveolar remodeling and the mechanisms of regulation during inflammation. Using novel holographic live cell imaging, we found that MC tryptase enhanced human bronchial and alveolar epithelial cell growth and shortened the cell division intervals. The elevated cell growth induced by tryptase remained in a pro-inflammatory state. Tryptase also increased the expression of the anti-apoptotic protein BIRC3, as well as growth factor release in epithelial cells. Thus, our data imply that the intraepithelial and alveolar MC release of tryptase may play a critical role in disturbing bronchial epithelial and alveolar homeostasis by altering cell growth–death regulation.

## 1. Introduction

Human mast cells (MCs) are immune cells strategically located at sites close to the external environment, such as the skin, gut and lungs, that play a crucial role in the first-line innate defense against bacteria, viruses and other foreign pathogens [1]. Upon activation, MCs release secretory granules, containing a wide range of pre-formed and de novo synthetized inflammatory mediators, such as histamine, serine proteases tryptase and chymase and cytokines. These mediators can be secreted in various ways, including anaphylactic degranulation and piecemeal degranulation, where small amounts of mediators from secretory granules are released continuously [2,3,4]. MCs are highly plastic cells, and their phenotypes are strongly influenced by signals from the local environment. In the healthy human lung, MCs are present in the large and small airway submucosa, pulmonary vessel walls and alveolar parenchyma. In chronic respiratory diseases, such as asthma and chronic obstructive pulmonary disease (COPD), MCs increase in numbers in these areas and are also located in tissues which normally have very few MCs, such as the bronchial epithelium [5,6,7,8,9]. MC granula, containing large amounts of serine protease tryptase, are involved in vital biological processes such as immunological responses, protection against toxins, wound healing and maintaining tissue homeostasis [10,11].

Airway and epithelial remodeling are pathological characteristics of chronic respiratory diseases, and involve alterations in the composition of the airway architecture in both the large and small airways as well as of the alveolar parenchyma. Eventually, these events lead to impaired airflow and ventilation. Although MCs are speculated to have an emerging role in disease progression [12,13], there is a limited understanding of the mechanisms underlying MC-induced chronic airway remodeling.

The bronchial and alveolar epithelium are under constant renewal and require tight regulation of cell growth, apoptosis and necrosis to maintain proper and fully functional barrier properties. Cell death signaling pathways can be activated upon stimuli such as infection and damage, and are important in regulating homeostasis within the airway epithelium. BIRC3 is a central anti-apoptotic protein and controls cell death homeostasis by regulating important signaling events in the caspase cascade [14]. Epithelial renewal and maintenance are also tightly regulated by growth factors (GFs), which are proteins that regulate cell growth, division and differentiation in various cell types [15]. For example, vascular endothelial growth factors (VEGFs), basic fibroblast growth factor (bFGF) and platelet-derived growth factor (PDGF) have been linked to airway remodeling and asthma pathology. These features are believed to be a result of chronic tissue damage, injuries and repair [15,16]. Despite this, the drivers of these events are not yet fully characterized nor understood.

We have previously shown that bronchial epithelial cells treated with tryptase upregulate the expression of the proliferation marker Ki67 and elevate mitochondrial activity. This was associated with enhanced proliferation; however, there was no effect on cell growth in tryptase-treated cells [17]. Although it has been suggested that tryptase induces mitogenic effects via protease activator receptor 2 (PAR-2) cleavage [10,18], the specific role of tryptase in cell growth control is yet to be determined. Specifically, the role of MC tryptase in tissue remodeling and cell growth in alveolar epithelial cells has not been studied, despite the high number of tryptase-positive MCs in the remodeled alveolar parenchyma in patients with chronic respiratory diseases. Hence, the overall aim of this study was to investigate the role of MC tryptase in airway remodeling and cell growth in human alveolar and bronchial epithelial cells. Since patients with asthma display chronic airway inflammation with viral-induced exacerbation, we additionally investigated these parameters in a pro-inflammatory and viral environment.

## 2. Materials and Methods

### 2.1. Mammalian Cells

Human bronchial epithelial cells (BECs), BEAS-2B (ATCC, Walkersville, MD, USA), and human alveolar epithelial cells (AECs), A549 from ECACC (Porton Down, Salisbury, UK), were cultured in RPMI-medium 1640 (Gibco, Paisley, UK, 61870-010) or Ham’s F12-medium (Gibco, Paisley, UK), respectively, with a supplement of 10% fetal bovine serum (FBS) and 1% penicillin–streptomycin (Life Technologies, Stockholm, Sweden). The cells were cultured in a humidified atmosphere at 37 °C in 5% CO_2_ until cell confluency reached 80–90%. All experiments on BECs and AECs were performed in starvation medium containing RPMI or F12 medium with 1% FBS and 1% penicillin–streptomycin. Primary human BECs were collected from patients with mild to moderate asthma who were not treated with inhaled corticosteroids for at least 3 months and had airway hyperresponsiveness to mannitol. Patients underwent bronchoscopy with endobronchial brush biopsies as part of the RECONSTRUCT study [19]. The study was approved by the Danish National Committee on Health Research Ethics (H-16043663) [19]. Cells were expanded and cultured according to previous validated protocols [20]. For the Ki67 and BIRC3 immunostaining, primary asthmatic BECs (cat#: 00194911) purchased from Lonza Bioscience, Switzerland, were used. Primary cells were seeded in collagen-coated T75 flasks, 6-well culture plates, 12-well culture plates or chamber slides. The supernatant, RNA and protein were harvested 6 and 24 h post-stimulation.

### 2.2. In Vitro Stimulations

Cells were stimulated with a highly purified human lung tryptase (Merck Millipore, Darmstadt, Germany) at a concentration at 0.5 μg/mL. As a viral mimic, 10 μg/mL TLR-3 agonist Poly(I:C) (polyinosinic:polycytidylic acid, purchased from InvivoGen, Toulouse, France was used and 10 μg/mL TLR-4 agonist, lipopolysaccharides (LPS) (Invivogen, France) or 2% cigarette smoke extract (CSE) were used to mimic pro-inflammatory conditions. For co-stimulation experiments, cells were pre-treated with tryptase for 3 h, followed by the addition of an indicated pro-inflammatory mediator. For assays blocking PAR-2, AECs were pre-exposed with antagonist I-191 (HY117793, MedChemExpress, Monmouth Junction, NJ, USA) for 30 min in 37 °C in 5% CO_2_, washed with PBS and thereafter stimulated with tryptase.

### 2.3. Cigarette Smoke Extraction

Cigarette smoke extract (CSE) was prepared according to Gellner et al. (2017) [21]. Research cigarettes (University of Kentucky, USA) were kindly provided by Prof. Arne Egesten, Lund University. Briefly, 8 cigarettes were marked with a permanent marker 23 mm from the end and attached to the apparatus containing 40 mL RPMI medium. Cigarette smoke extraction was performed inside a fume hood. The cigarettes were lighted and slow puffs (2–4 s) were made via a vacuum suction and repeated every 30 s until the marking of the cigarette was reached (total of 10–12 puffs per cigarette). A new cigarette was inserted into the holder and this procedure was repeated until all of the cigarettes had been smoked. This solution was defined as 100% CSE. The CSE was aliquoted and stored in a −20 °C freezer until use. To quantitate an appropriate dose for the cell experiments, dose–response experiments using the HoloMonitor and LDH assay analysis of the supernatants were performed.

### 2.4. Holographic Live Cell Imaging (HoloMonitor M4)

Bronchial and alveolar cell growth and cell division properties were studied using the HoloMonitor M4 live cell imaging system (Phase Holographic Imaging, Lund, Sweden). Cells were cultured in Sarstedt TC 6-well plates (Nümbrecht, Germany) 24 h prior to stimulation, at a cell concentration of 10,000 cells/cm^2^. A total of 7–10 focus points were taken per well/stimulation and images were captured every 15 min over 72 h, giving >2000 images per well and per repeat. To investigate the effect of tryptase on cell growth and division rate, data were based on three independent repeats and at least 15 focus points/stimulation. Cell growths are presented as a percentage of cell growth (relative to the starting number of cells) or number of cells at an indicated time point.

### 2.5. RNA Extraction and PCR Array

mRNA was collected and isolated 6 h post-protease stimulation using a RNeasy Mini Kit (QIAGEN, Hilden, Germany, 74106). The RNA concentration was measured using a Nanodrop 2000c from Thermo Scientific (Waltham, MA, USA) and transcribed to cDNA using an RT2 First Strand Kit (QIAGEN (Hilden, Germany), 330404). For PCR analysis, RT2 SYBR Green Rox Master Mix (QIAGEN, 330521), RT2 Profiler Array Assay (QIAGEN, PAHS-212Z) and RT2 primers BIRC3 (QIAGEN, PPH00326B), ACTB (housekeeping, QIAGEN PPH00073G) and HPRT1 (housekeeping, QIAGEN, PPH01018C) were used.

### 2.6. Luminex and ELISA

Protein release was detected in cell supernatants collected 6 and 24 h following treatment, using Luminex MAGPIX with Human Cell Growth Factor Fixed Panel (catalog ID: LKTM013) from R&D systems (Minneapolis, MN, USA) and ELISA DuoSet Kits from R&D Systems for bFGF, VEGF, Granzyme B, EGF and PDGF-AA, according to the manufacturer’s protocol.

### 2.7. Immunocytochemistry

Bronchial and alveolar epithelial cells were cultured in chamber slides (Merck Millipore, Darmstadt, Germany) until 70–80% confluency and then treated with tryptase for 24 h. Samples were fixated in 2% paraformaldehyde for 20 min and washed in PBS. For the staining, samples were permeabilized in 0.1% Tween-20 for 10 min and immunostained with primary antibody cIAP-2/BIRC3 (Thermo Fisher Scientific, Waltham, MA, USA, PA551700) diluted 1:50, or Ki67 1:300 (M7240, Dako, Denmark), and thereafter incubated with Alexa Fluor 488 (Invitrogen, Eugene, OR, USA) diluted 1:200. ProLong Glass antifade with NucblueTM (Thermo Fisher Scientific, Waltham, MA, USA, P36983) was used for mounting and nuclei visualization. A Nikon eclipse 80i combined with Nikon DS-QI1MC was used to capture images and Image-J was used for data analysis. Data are shown as positive pixels per quantified cell nuclei. The magnification of the immunostaining was visualized using a Nikon A1+ confocal microscope with a 20× Plan Apo objective, NA 0.75 (Nikon Instruments Inc., Tokyo, Japan), and acquired using NIS-elements, version: 4.60.02 (Laboratory Imaging, Nikon, Tokyo, Japan). All image captures were taken with identical settings.

### 2.8. Statistical Analysis

Statistical analysis was performed using GraphPad Prism 9.5.0 (GraphPad Software, La Jolla, CA, USA). Non-parametric Mann–Whitney U tests were used for comparison between two groups and the Kruskal–Wallis H test with Dunn’s multiple comparison test was used when comparing three groups or more. Results were considered to be statistically significant at *p* ≤ 0.05. * *p* ≤ 0.05, ** *p* ≤ 0.01, *** *p* ≤ 0.001, **** *p* ≤ 0.0001. The data are presented as mean ± SD.

## 3. Results

### 3.1. Mast Cell Tryptase Enhances Cell Growth in Alveolar and Bronchial Epithelial Cells

**Figure 1:**
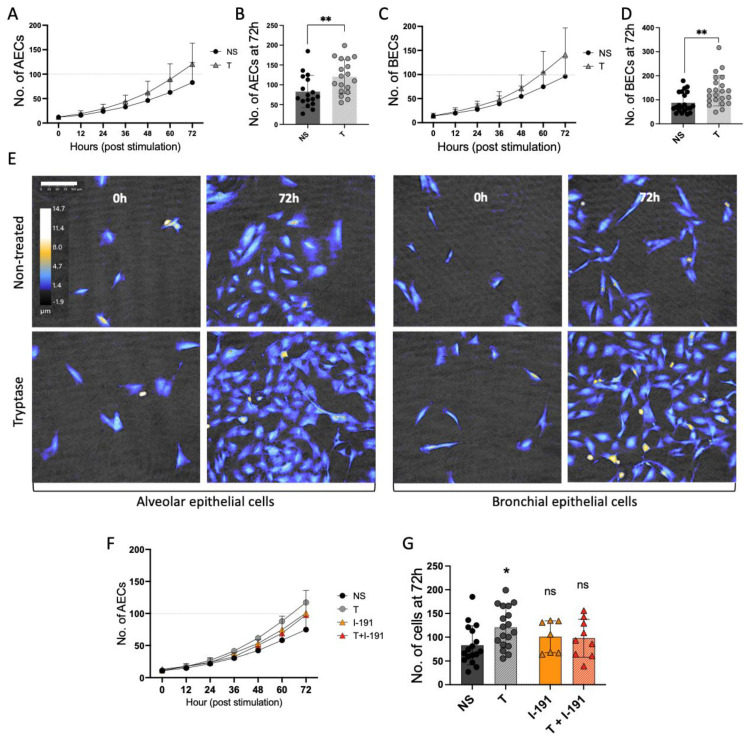
Holographic live cell imaging was used to investigate alveolar and bronchial cell growth. The growth curves show that MC tryptase increased the cell growth over time in both AECs (**A**) and BECs (**C**), compared to the NS cells. At 72 h, tryptase significantly enhanced the cell growth in alveolar and bronchial cells (**B**,**D**), in comparison to the NS cells. Data were based on three independent experiments and >15 focus points per group (≥4320 images/group across 72 h). Representative images (**E**) of alveolar (left) and bronchial (right) epithelial cells taken using the HoloMonitor M4 demonstrating cell numbers for NS and tryptase-treated cells at 0 h and 72 h. One image represents one focus point. Scale bar: 100 µm. The colors represent the cell height according to the color scheme in the top left image. The cell growth induced by tryptase was diminished in AECs when pre-treated with PAR-2 antagonist, in comparison to tryptase-treated cells alone, and there was no statistical difference compared to the NS AECs (**F**,**G**). Inhibitor analyses were based on two independent experiments and a total of ≥7 focus points per treatment (≥2016 images/group across 72 h). Data points for NS and tryptase-stimulated cells were based on the same three independent experiments, as presented in (**B**). Non-parametric Mann–Whitney tests were used for the comparison of the NS and tryptase-treated groups (**B**,**D**) and the Kruskal–Wallis H test with Dunn’s multiple comparison test was used for PAR-2 inhibitor analysis (**G**), * *p* < 0.05, ** *p* ≤ 0.01 using GraphPad Prism.

To better understand the implications of MC infiltration into the airways, this study focused on the role of MC proteases on alveolar and bronchial epithelial cell growth and homeostasis. By using holographic live cell imaging, we were able to perform thorough and detailed cell growth analysis, comparing the cell growth of untreated and tryptase-treated cells. Images (567 µm × 567 µm, 20× magnification) were captured every 15 min over 72 h, generating 288 images per focus point and ≥5 focus points per well, and stimulation was analyzed. In total, this resulted in ≥1440 analyzed images and approximately 375 analyzed cells per every single treatment and per repeat. As shown in the cell growth curves (Figure 1A,C), tryptase enhanced epithelial cell growth, both in AECs and BECs when compared to the non-stimulated (NS) cells. At 72 h, we found significantly elevated cell growth in the tryptase-treated cells compared to the untreated cells (Figure 1B,D) (AEC NS: 82 cells ± 41, AEC T: 120 cells ± 43, *p* = 0.009, BEC NS: 88 cells ± 42, BEC T: 138 cells ± 63, *p* = 0.003). Representative images were captured using the HoloMonitor M4 at the starting time (0 h) and 72 h post-treatment. Optimal cell viability and proliferation over time were observed in all groups using the HoloMonitor M4.

A PAR-2 inhibitor, I-191, was used to test whether the elevated cell growth induced by tryptase was a result of PAR-2 activation in the AECs. The results obtained using HoloMonitorM4 showed no statistical difference in cell growth when comparing NS alveolar epithelial cells with cells pre-stimulated with I-191 and then stimulated with tryptase (NS: 82 cells ± 41, T + I-191: 97 cells ± 40) (Figure 1F,G). As a control, we also analyzed cell growth in AECs treated with the inhibitor alone (100 cells ± 34), which did not induce a statistical difference in cell growth in comparison to NS.

### 3.2. Elevated Release of Growth Factors from Alveolar Cells Is Induced by Tryptase

**Figure 2:**
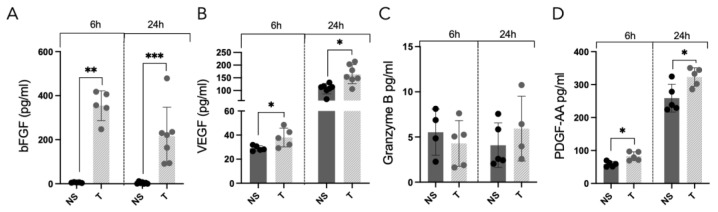
AECs treated with tryptase and the release of growth factors bFGF (**A**), VEGF (**B**), granzyme B (**C**) and PDGF-AA (**D**) were measured in supernatants using ELISA. Statistical significance between non-stimulated and tryptase-stimulated cells was tested using the Mann–Whitney test. Data represent the mean (±SD) and are based on ≥5 repeats. For Granzyme B, two data points were under the detection limit and are therefore not shown. * *p* < 0.05, ** *p* < 0.01, *** *p* < 0.001.

We have previously shown that tryptase induces GF release in bronchial epithelial BEAS-2B cells [22]; however, this has never been studied in AECs. AEC supernatants were analyzed for GFs, bFGF, VEGF, granzyme B and PDGF-AA. Our results showed that tryptase-treated cells had an increased release of bFGF (NS 6 h: 5.6 pg/mL ± 1.7, T 6 h: 354.4 pg/mL ± 67.4 *p* = 0.008, NS 24 h: 3.9 pg/mL ± 4.7, T 24 h: 216.2 pg/mL ± 131.0 *p* = 0.0006), VEGF (NS 6 h: 29.0 pg/mL ± 2.2, T 6 h: 37.9 pg/mL ± 7.8 *p* = 0.03, NS 24 h: 107.0 pg/mL ± 20.5, T 24 h: 164.1 pg/mL ± 37.5 *p* = 0.011) and PDGF-AA (NS 6 h: 58.7 pg/mL ± 7.9, T 6 h: 82.7 ± 13.2 *p* = 0.02, NS 24 h: 259.0 pg/mL ± 42.5, T 24 h: 323.1 pg/mL ± 28.0 *p* = 0.03), but not granzyme B (NS 6 h: 5.5 pg/mL ± 2.5, T 6 h: 4.3 pg/mL ± 2.5, NS 24 h: 4.1 pg/mL ± 2.5, T 24 h: 5.9 pg/mL ± 3.6), in comparison to NS cells (Figure 2A–D). Notably, the release of bFGF was the highest 6 h after tryptase treatment and decreased over time, whereas the levels of VEGF and PDGF-AA were higher at 24 h than at 6 h.

### 3.3. MC Tryptase Alters Cell Division Rate and Intervals in Epithelial Cells

**Figure 3:**
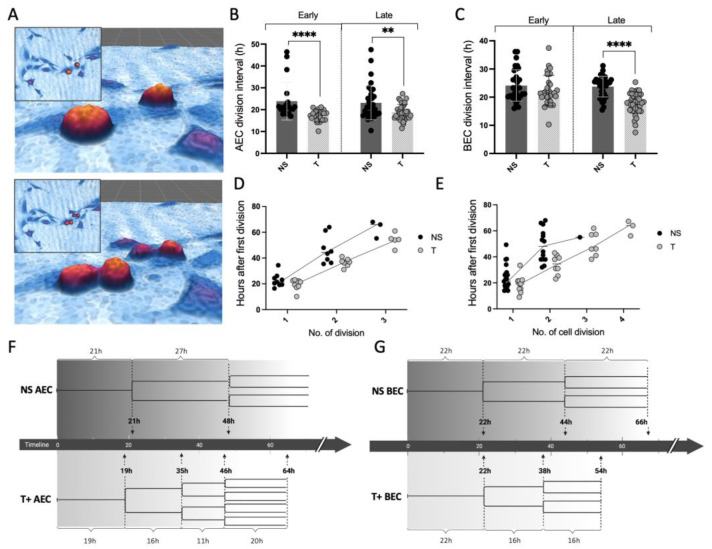
Tryptase altered cell division rates in alveolar and bronchial epithelial cells. Two-dimensional and three-dimensional images captured using HoloMonitor M4 illustrating cells’ pre- and post-cell division with a 15 min time difference (**A**). Tryptase induced significant shorter cell division intervals in both early and late phases of the experiment in AECs, compared to NS cells (**B**), whereas tryptase effect on BECs was not seen in the early phase but the later phase of the experiment (**C**). Values are shown as mean of three independent experiments and a minimum of 10 cells/group, and images were manually tracked throughout the experiment (144 pictures across 36 h). The analysis of individual cells showed a reduction in the time interval between cell divisions in tryptase-treated cells, both for AECs (**D**) and BECs (**E**). (**F**) (AECs) and (**G**) (BECs) demonstrate the obtained data from the HoloMonitorM4 in a schematic illustration of cell division properties of NS and tryptase-stimulated cells. (**D**–**G**) are based on tracking a minimum of 8 cells per image and stimulation across 72 h (in total more than 288 images/stimulation analyzed). Statistical significance between early and late responses was tested using the Mann–Whitney test. Data represent the mean (±SD). ** *p* < 0.01, **** *p* < 0.0001.

The effect of tryptase on the cell division rate in BECs and AECs was investigated using the HoloMonitor M4 (Figure 3A), whereby we also compared the time between cell divisions in the first phase of the experiment (0–36 h) with the later phase (36–72 h). The results showed that tryptase-treated AECs had a statistical reduction in cell division interval (i.e., fast cell cycle interval) already in the early phase of the experiment, when compared to NS cells (Figure 3B), and this effect remained in the later phase of the experiment (NS_early_: 23.9 h ± 8.7 and T_early_: 17.3 ± 2.7, *p* < 0.0001, NS_late_: 23.1 ± 7.8 and T_late_: 18.9 ± 3.5, *p* = 0.009). In BECs (Figure 3C), tryptase induced a statistically significant shortening of the cell division interval in the late phase of the experiment, in comparison to the NS cells, but not in the early phase (NS_early_: 24.1 h ± 5.6 and T_early_: 22.3 ± 3.6, *p* = 0.3, NS_late_: 23.8 ± 5.4 and T_late_: 18.1 ± 3.7, *p* < 0.0001).

To study the individual cell division patterns, single cells were tracked throughout the whole experiment (72 h) and the number of cell divisions as well as the time between cell divisions were calculated and compared between the stimulated and NS cells. AECs, stimulated with tryptase, had a gradually shorter time between cell division time points than NS cells (Figure 3D) (mean time of cell division interval: NS: first; 21 h, second; 27 h and tryptase stimulated: first; 19 h, second; 16 h, third; 11 h, fourth; 20 h). Only one single cell of the analyzed NS cells divided more than two times across the 72 h recorded experiments, whereas several of the tryptase-stimulated cells divided three (*n* = 7) and four times (*n* = 3). Tryptase-treated BECs (Figure 3E) showed similar, but less pronounced, tendencies compared to AECs to have shortened time division rates (mean time of cell division interval: NS: one; 22 h, two; 22 h, three; 22 h and tryptase stimulated: one; 22 h, two; 17 h, three; 17 h). Cell division trees illustrated the comparison between the division time points (Figure 3F,G).

### 3.4. Cell Death and Survival Regulatory Protein Expression Was Altered in Tryptase-Treated Cells

**Figure 4:**
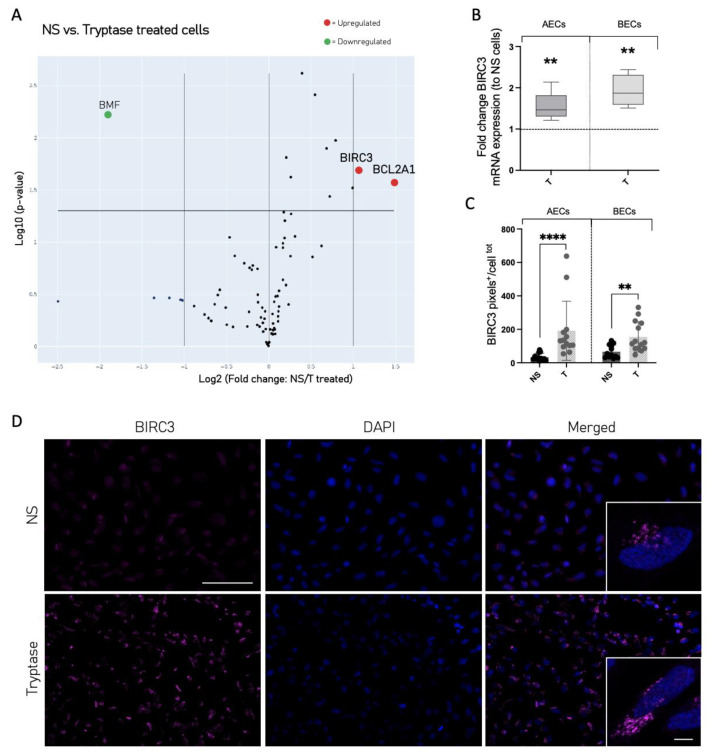
Tryptase induced pro-survival expression in alveolar and bronchial epithelial cells. A profiler gene array associated with cell survival and apoptosis genes was performed on BECs (based on three independent experiments) and the results are shown as a volcano plot (**A**). Upregulated gene expression of BIRC3 was confirmed via qPCR in both AECs and BECs (**B**) and was based on five independent experiments/cell types. Quantification of BIRC3 protein expression (pixels^+^/cells^total^) in AECs and BECs (positive cells were analyzed in ≥4 fields of view with 10× magnification in three independent experiments) using fluorescence immunocytochemistry and Image-J (**C**). Representative micrographs of immunofluorescently stained BIRC3 (FITC, magenta) and nuclei (DAPI, blue) in BEAS-2b cells stimulated with tryptase and compared to NS cells (**D**). Scale bar in (**D**), upper left: 100 µm, magnifications: 10 µm. Statistical significance between non-stimulated and tryptase-stimulated cells was tested using the Mann–Whitney test. Data represent the mean ± SD, ** *p* < 0.01, **** *p* < 0.0001.

One factor which can promote tissue remodeling is the dysregulation of the cell death and survival balance. Therefore, we wanted to study weather tryptase may influence the cell death–survival regulation. Data from a RT2 profiler gene array associated with cell survival and apoptosis revealed that tryptase enhanced the gene expression of two anti-apoptotic genes, BIRC3 and BCL2A1, in BECs (Figure 4A). The gene expression of BIRC3 was further confirmed using qPCR and showed that both AECs and BECs treated with tryptase had a significantly higher gene expression of BIRC3 (AEC: 1.5 ± 0.4, *p* = 0.008, BEC: 1.9 ± 0.4, *p* = 0.008, data represent fold change in NS cells) (Figure 4B). The quantification of BIRC3 protein expression (i.e., pixel intensity) was performed using immunocytochemistry and Image-J (Figure 4C,D) on both AECs and BECs. The results confirmed the upregulation of BIRC3 protein expression in tryptase-treated cells in comparison to NS cells (AEC NS: 34.1 pixels^+^/cell^total^ ± 22.4 and AEC T: 191.3 pixels^+^/cell^total^ ± 176.8 pixels^+^/cell^total^, *p* = 0.004, BEC NS: 66.7 pixels^+^/cell^total^ ± 38.9 and BEC T: 155.1 pixels^+^/cell^total^ ± 90.6, *p* = 0.003).

### 3.5. The Mitogenic Effect of Tryptase Remained in Pro-Inflammatory Environments

**Figure 5:**
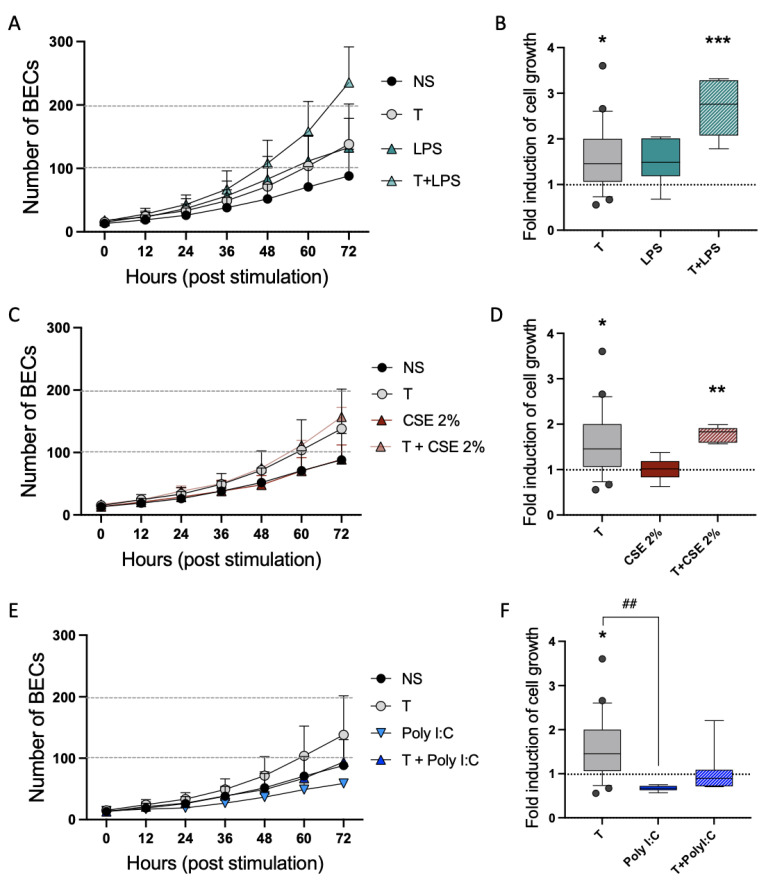
Elevated cell growth induced by tryptase remained in a viral and pro-inflammatory environment. Holographic live cell imaging was performed to study the cell growth curves of BECs. Cells were either NS, stimulated with the pro-inflammatory mediator LPS (**A**,**B**), cigarette smoke extract (CSE, **C**,**D**) or viral mimic poly:IC (**E**,**F**) or co-stimulated with tryptase. The fold induction of cell growth at 72 h was compared to NS cells for statistical analysis (**B**,**D**,**F**). Data represent analysis from ≥5 images per time point and pro-inflammatory or viral stimuli. Data points for NS and tryptase-stimulated cells were based on the same three independent experiments as presented in Figure 1D. Statistical significance between groups was tested using the Kruskal–Wallis H test with Dunn’s multiple comparison test. Data represent the mean ± SD, and fold changes are relative to the NS cells. * *p* < 0.05, ** *p* < 0.01, *** *p* < 0.001 compared to NS, ## *p* < 0.01 comparison between T and PolyI:C.

Since epithelial cells from patients with chronic respiratory disease show increased sensitivity to viral and pro-inflammatory stimuli [20,23], the effects on cell growth of tryptase on BECs were investigated after stimulation with LPS (Figure 5A), CSE (Figure 5C) and polyI:C (Figure 5E). Tryptase induced increased cell growth compared to NS cells in all experiments (as also shown in Figure 1D). Both T + CSE (1.8 ± 0.2, *p* = 0.005) and T + LPS (2.7 ± 0.6, *p* = 0.0001) showed higher cell growth compared to NS cells, indicating that the mitotic effect induced by tryptase is still present in the presence of pro-inflammatory mediators. PolyI:C-stimulated cells (0.7 ± 0.06) showed significantly lower cell growth compared to tryptase-stimulated cells alone (1.6 ± 0.7, *p* = 0.002) and did not increase in combination with tryptase compared to NS cells (*p* = 0.9), indicating less effect of tryptase on cell growth in the presence of viral mimic polyI:C.

### 3.6. Tryptase Enhanced Protein Expression of Ki67 and BIRC3 as Well as Increased Growth Factor Release in Primary Human Bronchial Epithelial Cells from Patients with Asthma

**Figure 6:**
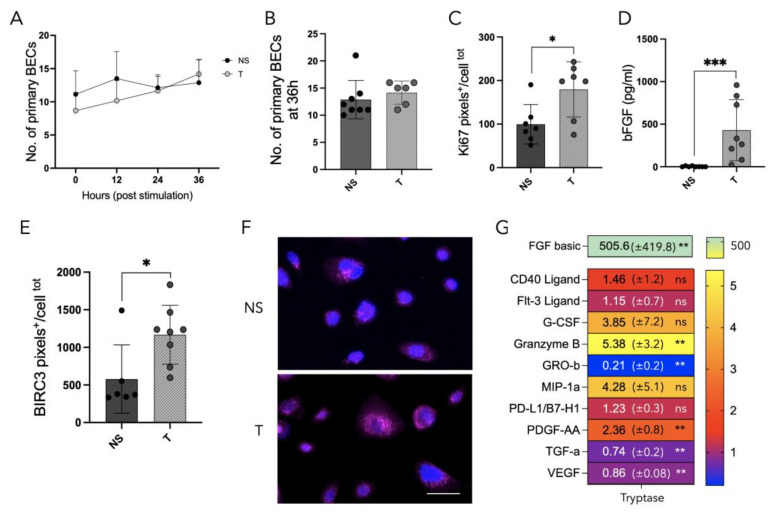
Mast cell tryptase impact on primary bronchial epithelial cells from patients with asthma. Holographic live cell imaging was performed for cell growth evaluation and did not show a defined difference in cell growth between NS cells or tryptase-treated primary BECs across 36 h (**A**), or at 36 h (**B**). A total of 7–8 focus points per group generating a total of 864 images/group were analyzed. Image analysis of ICC micrographs of the proliferation marker Ki67 revealed increased protein expression of tryptase-stimulated primary BECs, in comparison to NS cells (**C**). Positive cells were analyzed in 7 fields of view with 10× magnification. Supernatants were analyzed using ELISA and revealed an increased release of bFGF in tryptase-stimulated primary BECs compared to NS cells (*n* = 8) (**D**). Image analysis of immunofluorescently stained BIRC3 in primary BECs (**E**,**F**) showed significant increase in positive pixels/cells^total^ in tryptase-treated cells, in comparison to NS cells; ≥6 fields of view with 10× magnification/stimulation. Scale bar: 30 µm. Luminex analysis revealed that tryptase has a strong impact on the release of several growth factors in primary BECs (**G**), e.g., bFGF, granzyme B, GRO-β, MIP-1a and TGF-α. Luminex data are presented as fold change in NS cells from five asthmatic subjects. Statistical significance between non-stimulated and tryptase-stimulated cells was tested using Mann–Whitney test. Data represent the mean ± SD. * *p* < 0.05, ** *p* < 0.01, *** *p* < 0.001, (ns: non-significant).

To further understand the role of intraepithelial MCs in a more translational setting, the experiments performed on cell lines were repeated in primary human BECs from patients with asthma. First, the role of tryptase on the cell growth of primary BECs was assessed using holographic live cell imaging and no significant difference was observed at 36 h, comparing NS cells (13 ± 4) with tryptase-treated cells (14 ± 2, *p* = 0.17; Figure 6A,B). Due to different cell growth patterns and the sensitivity of primary cells compared to cells from commercial cell lines, the cell growth experiment was limited to 36 h. The lack of difference in cell growth that was observed at this time point was consistent with our previous findings in BEAS2B cells [17]. Immunocytochemistry was used to investigate whether tryptase influenced proliferation by measuring Ki67 protein expression, which was observed in Beas2b [17]. Image analysis revealed a statistical increase in Ki67 expression in tryptase-stimulated primary BECs (179.6 pixels^+^/cell^total^ ± 63.2) when compared to NS cells (100 pixels^+^/cell^total^ ± 45.3, *p* = 0.03) (Figure 6C).

Furthermore, bFGF release was analyzed in primary cells stimulated with tryptase (Figure 6D), and similarly to previous observations in bronchial and alveolar cell lines, tryptase significantly increased the release of bFGF (430.0 pg/mL ± 358.5, *p* = 0.0002) in primary BECs as well, when compared to NS cells (3.7 pg/mL ± 4.9).

The anti-apoptotic marker BIRC3 was fluorescently stained (Figure 6E) and image analysis revealed an upregulation of BIRC3 positive pixels in primary BECs as well when comparing NS cells (578.0 pixels^+^/cell^total^ ± 453.9) with tryptase-stimulated cells (1168.0 pixels^+^/cell^total^ ± 391.2, *p* = 0.03).

Lastly, since the alteration of GF release in bronchial and alveolar epithelial cell lines was induced by tryptase, the release pattern of a broader panel of human GFs was investigated using a multiplex Luminex assay of primary BEC supernatants. The data revealed that tryptase influenced the release of GFs in primary BECs when compared to NS cells (Figure 6G). Among the significantly upregulated proteins released in tryptase-stimulated primary BECs were bFGF, granzyme B and PDGF-AA, and the significantly downregulated released proteins were GRO-β, TGF-α and VEGF. The data are presented as fold change in NS cells. Notably, in contrast to our cell lines, the release of VEGF in primary BECs was decreased in tryptase-stimulated cells, compared to NS cells.

## 4. Discussion

In the current study, we investigated the role of mast cell (MC) protease tryptase on fundamental homeostatic cell growth responses in AECs and BECs and whether these effects are affected during pro-inflammatory or viral conditions. Our results show that tryptase enhances cell growth and the shortening of time intervals between cell divisions, in both AECs and BECs. We also found that tryptase alters cell growth and death regulation by increasing GF release and anti-apoptotic expression in both alveolar and bronchial cell lines and in primary bronchial epithelial cells. Thus, our results imply that tryptase plays a major role in regulating the local environment with respect to enhanced epithelial cell growth and survival. To our knowledge, this is the first time that the mitogenic effects of tryptase have been functionally studied from an alveolar perspective using live cell imaging, which gives a highly detailed understanding of both cell growth and cell division properties.

Airway remodeling (AR) and chronic inflammation are the main features of many airway diseases, which eventually lead to a loss of lung function. AR affects both small and large airways and includes numerous different structural changes, such as the thickening of epithelial basement membrane, fibrosis, goblet cell metaplasia, and hyperplasia of airway smooth muscle cells, which together result in airway wall thickening and airflow obstruction [24,25,26]. Increasing the amount of data indicates inflammation and tissue remodeling of the alveolar parenchyma in asthma [27]. Furthermore, alveolar parenchyma is a major site of remodeling in COPD and IPF [28]. In uncontrolled and severe asthma, COPD, IPF and cystic fibrosis, there is a drastic increased population of MCs expressing both tryptase and chymase in the airway and alveolar tissue [5,29,30,31]. Major focus has been placed on the role of MCs in the inflammatory response in the submucosa of the large airways; however, functional information with regard to the implication of MC infiltration into the alveoli and bronchial epithelium is still lacking. Here, MC infiltration may induce and contribute to airway remodeling and chronic inflammation.

Several studies, from us and others, have previously reported that tryptase induces mitogenic activities in vitro, e.g., increased metabolic activity, Ki67 expression and [3H] thymidine incorporation, which are indications of proliferative effects, migration and increased gap closure in BECs [17,32,33], but, as far as we are aware, tryptase impact on proliferation has not been functionally shown in BECs nor has it been investigated in AECs. We found that tryptase functionally enhanced cell growth in both AECs and BECs when compared to NS cells in cell lines. Although we aimed to confirm these findings in primary BECs obtained from patients with asthma, we did not find a clear functional effect on cell growth in tryptase-treated primary BECs. However, we found a statistical upregulation of Ki67 in tryptase-treated primary cells when compared to NS cells. These results suggest that tryptase may induce some proliferative effects in primary cells, via Ki67, but more experiments with larger patient cohorts are needed to study the functional role of tryptase on cell growth in patients. In future studies, a live cell imaging experimental set up may be optimized due to differences in growth patterns between cell lines and primary cells and it may be relevant to compare cells from healthy and diseased subjects. Additionally, it would be interesting to study the effect of tryptase on patient cells during cell growth in fully differentiated human BECs. Studying whether short-term and repeated long-term stimulation with tryptase may affect epithelial cell growth in differentiated air–liquid interface (ALI) cultures is needed.

To investigate whether the cell growth alterations, which were found in the cell lines, were mediated via PAR2, inhibitor I-191 was used. Although a small dampening in cell growth could be observed with I-191, the effect was not significant. This might indicate that PAR-2 signaling is less important for cell growth in AECs compared to BECs, where the effect is more pronounced [33]. In a recent study by Zhao et al., a small but significant effect of tryptase on cell viability was found, as well as an increased release of IL-1RA, CCL27, amphiregulin (AREG), CXCL11 and IGFBP-6 in primary small airway epithelial cells [34]. This is in line with our data and supports the notion that tryptase may have important effects on airway epithelial remodeling. Zhao et al. also showed that chymase induced several significant effects, important in epithelial remodeling, in primary small airway cells [34], which could be of great importance since chymase-positive MCs increase in chronic respiratory diseases [5,29,35].

Furthermore, we wanted to investigate whether the cell division pattern was altered by tryptase. Interestingly, we found that tryptase-treated BECs had a shorter cell division interval in the later phase of the experiment, i.e., after the first and second cell division, whereas in alveolar epithelial cells, tryptase had a faster action and had induced a shorter cell division rate already at an earlier time point. Interestingly, these changes persisted up to 72 h. We have previously shown that the majority of the enzymatic activity of tryptase is lost after approximately 6–12 h post-stimulation [17]. We have also shown that the cell growth induced by tryptase is normalized using the serine protease inhibitor AEBSF in bronchial epithelial cells [22]. Our present data indicate that the long-term effects of tryptase on cell division are not likely to be induced only by direct enzymatic activity but by other cellular changes induced by tryptase, yet to be elucidated. As illustrated in Figure 3G, tryptase-treated AEC divided up to four times across the 72h experiment, whereas untreated cells mainly divided two times across the same time period. This is also interesting from the perspective of tumor progression. In several solid tumors, such as gastric and pancreatic cancers, MCs are pro-tumorigenic and are associated with poor diagnosis, although contradictory results have been shown in other cancer types, e.g., breast cancer, where MCs, in contrast, appear to be antitumorigenic. The dual role of MCs in tumor progression is conflicting, and little is known regarding their role in tumor progression; however, this appears to be contingent on their microlocalization and tumor subtype [36]. The insights regarding the tryptase mitogenic effect on different kinds of cell types may, however, be highly relevant in regard to cancer research, for example.

A broad range of cytokines and GFs, e.g., basic FGF and PDGF-AA, act as central factors in epithelial and alveolar remodeling and respiratory disease progression due to their potent mitogenic and chemoattractive properties. Although GFs are crucial for tissue maintenance and survival, an overexpression or dysregulation of GFs have detrimental roles and are commonly seen in chronic respiratory diseases, including asthma, COPD and IPF. The underpinning mechanisms of altered GF homeostasis in alveolar parenchyma and airways are not fully understood [15,26,37,38,39,40]. We hypothesized that the MC infiltration into airway tissue seen in disease conditions is involved in tissue remodeling directly (mitogenic properties in epithelial cells and release of granula containing GFs) and indirectly, via the alteration of normal GF balance in airway tissue by inducing GF release in other structural cells. Our group has previously reported an elevated release of several GFs, including bFGF, VEGF and PDGF-AA, in tryptase-treated BECs [33]. Since MC_TC_s infiltrate the alveolar parenchyma in many respiratory diseases, which is also strongly affected by remodeling, we aimed to investigate the tryptase effect of GF release in alveolar epithelial cells. In the present study, we demonstrated, for the first time, that tryptase significantly increases GF release in both primary BECs from patients with asthma and also in AECs. Luminex data from tryptase-treated primary cells revealed that tryptase shifted the GF profile since it upregulated several GFs (bFGF, granzyme B, PDGF-AA and MIP-1a) and decreased others (e.g., GRO-β and TGF-α) when compared to non-stimulated cells. These findings again emphasize the detrimental role of MCs in chronic respiratory disease pathogenesis, since these GFs have strong cell growth and chemotactic effects on multiple structural cell types [15,37,41,42].

The cellular inhibitor of apoptosis proteins (cIAP)2, also known as BIRC3, is an anti-apoptotic protein, which is highly important in the control of cell death–survival regulation. In recent years, it has particularly been highlighted for its contribution in cancer progression by inhibiting apoptosis and thus promoting uncontrolled cancer cell proliferation and tumor survival. BIRC3 exerts pro-survival functions by regulating caspases and pro-caspases [14,43,44]. Du et al. showed that levels of BIRC3 were significantly increased in sputum in a cohort of asthma patients, and the authors suggest the involvement of BIRC3 in the pathogenesis of asthma [45]. While they show strong links, the mechanistic understanding of increased BIRC3 in sputum from patients with asthma is not known. However, in the present report, our data reveal that MC protease tryptase enhances the expression of BIRC3, in both alveolar and primary bronchial epithelial cells. Our data raise the possibility that intraepithelial MCs and the release of tryptase are involved in the increased epithelial expression of BIRC3 present in the asthma cohort. In the study by Zhao et al., no increased expression of anti-apoptotic mediators upon tryptase stimulation in small airway epithelial cells was observed [34]. One important explanation could be the use of epithelial primary cells vs. cell lines; however, the induction of BIRC3 was confirmed in primary bronchial epithelial cells in the present study. An important difference between our study and the results from Zhao et al. is the use of recombinant β tryptase vs. human lung tryptase. MC tryptase is a tetrameric enzyme comprising a combination of α and β (βI-III) subunits. The effect of different types of tryptase on epithelial cells has not been studied, but could potentially give rise to variances in cellular function. This modified cell death–survival regulation may be important with regard to the altered cell growth control induced by tryptase and may have implications on epithelial remodeling.

Since patients with asthma show augmented chronic airway inflammation and viral-induced exacerbations, we further wanted to study the consequence of tryptase on airway cell growth in pro-inflammatory and viral environments. We used three agents strongly associated with the induction of airway inflammation and viral responses: the viral mimic polyI:C, LPS and cigarette smoke extract (CSE). Our results showed that the elevation in BEC growth induced by tryptase remained in a pro-inflammatory environment (LPS and CSE). PolyI:C-stimulated cells showed a tendency toward poorer cell growth that, unlike LPS and CSE, did not increase in the presence of tryptase. Interestingly, viral infections are the main cause of exacerbations and epithelial damage in asthmatic patients, which also show impaired healing capacity in the bronchial epithelium [46,47]. However, more experiments are needed to confirm and verify these data. Without any significantly reducing effects on cell growth by the noxious stimulus itself, it is difficult to draw any definite conclusions from these experiments, and more studies are needed on the functional aspects of tryptase during viral infections.

## 5. Conclusions

In this study, our results reveal that tryptase exerts the ability to induce cell growth and the shortening of cell division intervals, and alter anti-apoptotic regulation and growth factor release in both human alveolar and bronchial epithelial cells. In addition, we show that tryptase-treated primary bronchial epithelial cells from asthma patients enhanced the protein expression of the anti-apoptotic protein BIRC3, altered growth factor release and elevated the expression of the proliferative marker Ki67, although no functional difference was observed. Taken together, our findings imply that intraepithelial MCs and their release of tryptase may be key players in airway remodeling by disturbing normal cell growth–death homeostasis and hence promote epithelial cell growth and survival and possibly other structural cells in the lung tissue as well. Apart from advancing the concept of the role of MCs in chronic respiratory diseases, these observations may provide important insights into how to improve treatment strategies by targeting tryptase.

## Data Availability

Data available upon request due to restrictions, e.g., privacy or ethical. The data presented in this study are available upon request from the corresponding author.
